# Genetic variation of glycophorins and infectious disease

**DOI:** 10.1007/s00251-022-01280-7

**Published:** 2022-10-12

**Authors:** Edward J. Hollox, Sandra Louzada

**Affiliations:** 1 Department of Genetics and Genome Biology, University of Leicester, Leicester, UK; 2 Laboratory of Cytogenomics and Animal Genomics, Department of Genetics and Biotechnology, University of Trás-Os-Montes and Alto Douro, Vila Real, Portugal; 3 BiolSI - Biosystems & Integrative Sciences Institute, Faculty of Sciences, University of Lisboa, Lisbon, Portugal

**Keywords:** Genetic variation, Glycophorins, Malaria, Infectious disease, Copy number variation

## Abstract

Glycophorins are transmembrane proteins of red blood cells (RBCs), heavily glycosylated on their external-facing surface. In humans, there are four glycophorin proteins, glycophorins A, B, C and D. Glycophorins A and B are encoded by two similar genes *GYPA* and *GYPB*, and glycophorin C and glycophorin D are encoded by a single gene, *GYPC*. The exact function of glycophorins remains unclear. However, given their abundance on the surface of RBCs, it is likely that they serve as a substrate for glycosylation, giving the RBC a negatively charged, complex glycan “coat”. *GYPB* and *GYPE* (a closely related pseudogene) were generated from *GYPA* by two duplication events involving a 120-kb genomic segment between 10 and 15 million years ago. Non-allelic homologous recombination between these 120-kb repeats generates a variety of duplication alleles and deletion alleles, which have been systematically catalogued from genomic sequence data. One allele, called DUP4, encodes the Dantu NE blood type and is strongly protective against malaria as it alters the surface tension of the RBC membrane. Glycophorins interact with other infectious pathogens, including viruses, as well as the malarial parasite *Plasmodium falciparum*, but the role of glycophorin variation in mediating the effects of these pathogens remains underexplored.

## Glycophorin genes and function

**Fig. 1:**
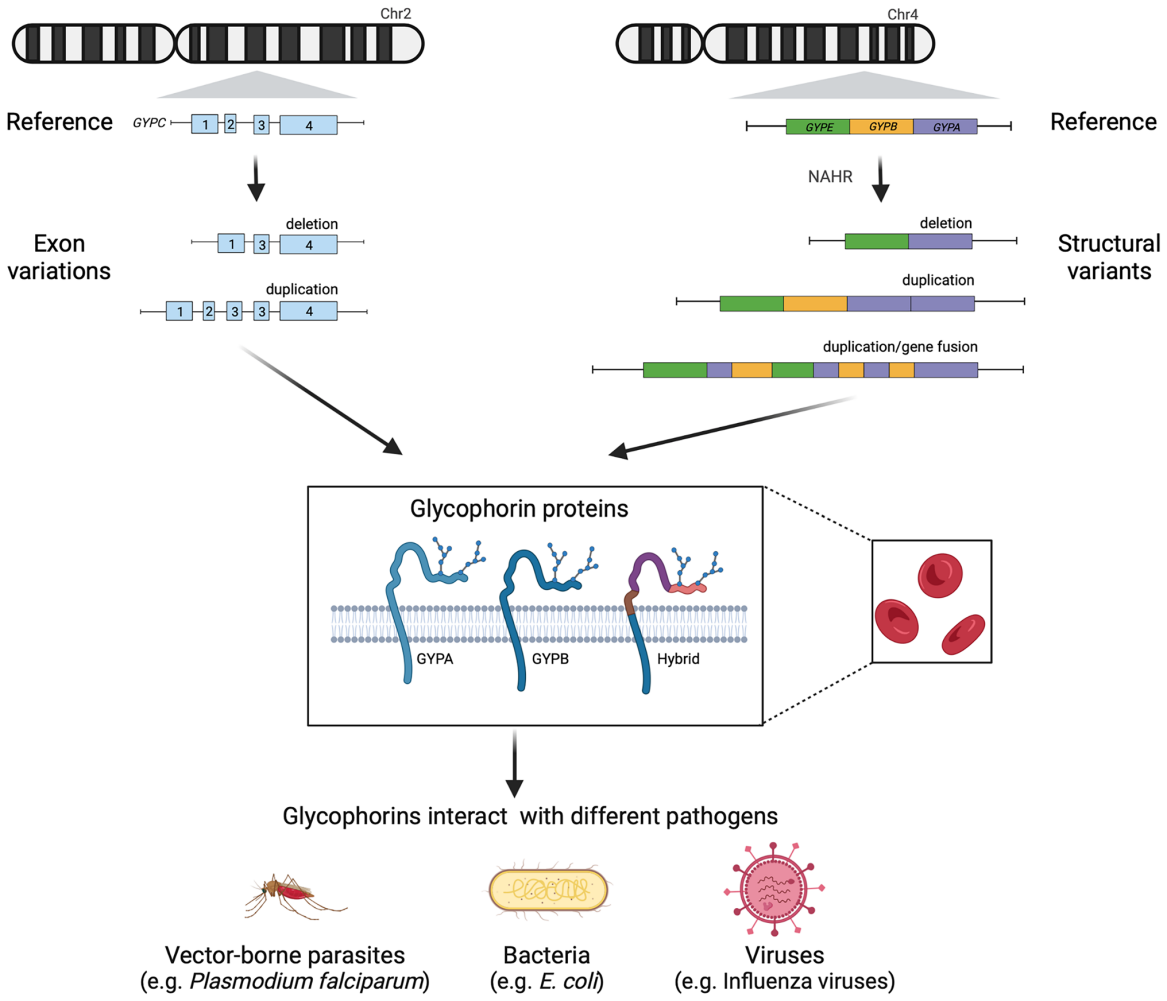
Summary of the role of glycophorins and infectious disease. Overview of the central concepts discussed in this review. Structural variation affects two distinct loci carrying the *GYPC* gene and *GYPA*/*GYPB*/*GYPE*. Different variants encode distinct glycophorin variants on the red blood cell surface. These glycophorins interact with a variety of different pathogens, including viruses, bacteria and malaria. Created with Biorender.com

Glycophorins are transmembrane proteins of red blood cells (RBCs), heavily glycosylated on their external-facing surface. In humans, there are four glycophorin proteins, glycophorins A, B, C and D. Glycophorins A and B are encoded by two similar genes *GYPA* and *GYPB*, and glycophorin C and glycophorin D are encoded by a single gene, *GYPC*, which is not related to *GYPA/GYPB*. Glycophorin C and glycophorin D differ due to different translational start sites on the *GYPC* transcript (Le Van et al. 1987). A gene annotated as *GYPE*, which is very similar to *GYPA* and *GYPB*, is transcribed, but no protein product for glycophorin E has been detected; therefore, *GYPE* is likely to be a pseudogene (Fig. 1, Vignal et al. 1990). Glycophorins have been characterised as carrying the antigens for several human blood groups. Glycophorins A and B carry the MN and Ss blood groups, and glycophorin C carries the Gerbich blood group system (Daniels 2008; Lopez et al. 2021). Rare individuals without glycophorin A (En), glycophorin B (S- s- U-) or both (M^k^) have been identified by the absence of particular blood groups carried by these proteins. Individuals who lack glycophorin B or glycophorin A are healthy (Tokunaga et al. 1979), so the exact function of these glycophorins remains unclear. Glycophorin C has been shown to have a role in maintaining the biconcave discoid shape of the RBC (Reid et al. 1987). Given their abundance of glycophorins on the surface of RBCs, it is likely that they also serve as a substrate for glycosylation, giving the RBC a negatively charged, complex glycan “coat” allowing circulation without adherence to other cells or walls of blood vessels.

## Evolution of glycophorin genes in primates

**Fig. 2:**
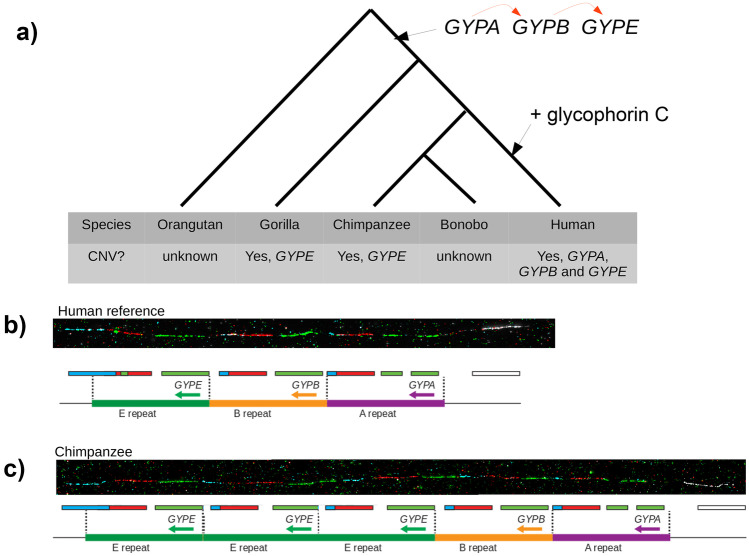
Evolution of glycophorins in great apes. **a** The tree shows the phylogeny of great apes, with branches annotated with the changes in glycophorin genes along the branches. **b** Fibre-FISH representative image of the human glycophorin region showing the reference haplotype. 120-kb repeats carrying *GYPE*, *GYPB* and *GYPA* are represented by coloured bars green, orange and purple, respectively. Each one of the genes was identified by a specific FISH pattern using region-specific fosmid clones (details in Louzada et al. 2020). **c** Structural variation in the glycophorin region in chimpanzee revealed by fibre-FISH, highlighting the presence of three copies of GYPE (fibre-FISH details in Louzada et al. 2020)

Primates, and other mammals, have a single *GYPA* gene, with the exception of bonobos, chimpanzees, gorillas and humans, which all have three related genes (*GYPA*, *GYPB* and *GYPE*) (Rearden et al. 1993), sharing about 97% identity. These three genes were generated by two duplication events after divergence of orangutans but before divergence of gorillas from the human lineage (about 10–15 MYA) (Fig. 2a; Kudo and Fukuda 1990; Rearden et al. 1993). There is no evidence for duplication of *GYPC*, as all primates have a single *GYPC* gene (Wilder et al. 2009). However, the translation initiation codon for glycophorin C appears to be specific to humans, with the translation initiation codon for glycophorin D conserved across apes. Glycophorin C is therefore a human-specific protein, with glycophorin D being present in all apes (Wilder et al. 2009).

## Structural variation of glycophorin genes

The glycophorin genes A, B and E are on approximately 120-kb tandemly arranged repeats on chromosome 4 (Fig. 2b), and, because of this, are prone to rearrangements driven by recurrent non-allelic homologous recombination (NAHR) events. These events can be either deletions or duplications, and involve either *GYPA*-*GYPE*, *GYPA*-*GYPB* or *GYPB*-*GYPE* as partners. More complex events can be generated, most likely the result of a series of individual NAHR events. If the complex events involve the regions where the glycophorin genes are encoded, then fusion genes can be formed from different exons of *GYPA*/*GYPE* and *GYPB*. Many of these variants were initially detected as novel, rare, blood groups (Daniels 2008). Analysis of the molecular genetic basis of particular rare blood groups (e.g. some alleles with the S- s- blood group, Willemetz et al. 2015) has shown that gene conversion, where a region from one gene is “copied and pasted” into another, is a further source of genetic variation. Because *GYPC* is in a single copy region on chromosome 2, the gene is not prone to extensive complex structural variation; however, the Gerbich negative blood types are caused by small deletions of exon 2 (Ge2), exon 3 (Ge3) or both exons 2 and 3 (Ge4) and the Gerbich Lsa antigen is caused by a duplication or triplication of *GYPC* exon 3 (Jaskiewicz et al. 2018).

Genome sequence data has allowed a systematic cataloguing of structural variants across the region (Leffler et al. 2017). Many have been validated by fibre-FISH, breakpoint PCR, and some have been shown to underlie blood group variation (Louzada et al. 2020). The sizes of the observed duplications and deletions usually correspond to loss or gain of one, or sometimes two, repeat units of ~ 120 kb each. The most complex structural variant yet identified is DUP4, which is the molecular basis of the Dantu NE blood group. This is partial duplication/triplication and generates loss of *GYPB* but a duplication of *GYPE* and three copies of a novel *GYPB-GYPA* fusion gene which is expressed on the RBC surface (Leffler et al. 2017; Algady et al. 2018). In contrast to structural variants that cause changes in copy number, a systematic exploration of gene conversion variants and inversion variants in the region is lacking. Given the challenges in mapping short sequence reads to duplicated regions such as the glycophorin A-B-E region, accurate long sequence reads will be needed to robustly distinguish gene conversion events from sequence read mis-mapping.

Compared to humans, little is known about structural variation in primate glycophorins. Genome assemblies using long-read sequence data give an indication of at least one structural arrangement of the region, for example the latest bonobo assembly (Mao et al. 2021, panPan3) shows the same glycophorin arrangement as humans. However, the genome region containing glycophorin A-B-E is currently incompletely assembled in the most recent gorilla assembly (ggor6) and chimpanzee assembly (Clint_PTRv2), presumably because of its highly duplicated structure. Although the A-B-E genomic structure has been confirmed in gorillas (Xie et al. 1997), we have observed a gorilla with a total of four glycophorin genes using fibre-FISH, though we were unable to determine whether the extra glycophorin gene was *GYPA*, *GYPB* or *GYPE* (Louzada, Hollox and Yang unpublished). It is known that *GYPE* is polymorphic in copy number in gorillas, being completely absent in 9/16 individuals (~ 56%) (Rearden et al. 1993), so the extra gene we observe is likely to be *GYPE*. In an early chimpanzee reference genome (panTro2), three *GYPE* genes were annotated (Ko et al. 2011), and this arrangement confirmed using fibre-FISH (Fig. 2c). It is likely that other genes, beyond *GYPE*, will be copy number variable in chimpanzees and gorillas, but a comprehensive study is needed.

## Genotyping the variation in glycophorin genes

Although genome sequencing is becoming cheaper and more cost effective, there is still an important role for methods designed to genotype structural variants by PCR, particularly for limited samples or in situations with limited resources. For DUP4, methods involving PCR amplification of *GYPB-GYPA* and *GYPA* followed by restriction enzyme digestion and gel electrophoresis to distinguish the genes (Leffler et al. 2017) or breakpoint-specific PCR (Algady et al. 2018) have been developed. Designing a PCR spanning the SV breakpoint is challenging because PCR primers are designed to be specific not only to the allele but the paralogue as well. However, for other variants, in particular GYPB deletion alleles, breakpoint-specific PCRs and PCR-based paralogue ratio tests have been developed (Lane et al. 2020; Algady et al. 2021; Amuzu et al. 2021).

For genotyping single nucleotide variation, mis-mapping of short sequencing reads between paralogues can limit accuracy, particularly in regions where gene conversion alleles have occurred. Similarly, paralogues need to be distinguished in PCR approaches by carefully validating the paralogue-specificity of PCR primers, to ensure the correct locus is genotyped. As for structural variation, long read sequencing will make accurate genotyping of these duplicated regions more reliable, and allow for improvements in haplotype phasing of variants.

## Glycophorins in malaria

Both glycophorin A and glycophorin B act as receptors EBA-175 and EBL-1 on the surface of *Plasmodium falciparum*, the parasite responsible for malaria in Africa. Glycophorin C also interacts with *P. falciparum* through its EBA-140 receptor (Wassmer and Carlton 2016). The DUP4 structural variant, encoding the Dantu blood group, has been shown to be protective against severe malaria, with homozygotes showing 74% protection against severe malaria (Field et al. 1994; Leffler et al. 2017). Furthermore, in a village-based non-hospital setting with endemic *P. falciparum* malaria, DUP4 has been shown to be associated with higher haemoglobin levels, likely reflecting DUP4 protection against malarial anaemia (Algady et al. 2018). DUP4 protects against malaria not by altering ligand-receptor interactions with *P. falciparum*, but by increasing the RBC surface tension preventing *P. falciparum* invasion (Kariuki et al. 2020).

Despite functional evidence showing that RBCs completely lacking glycophorins A and B are partially resistant to *P. falciparum* invasion (Hadley et al. 1987), there is no genetic evidence suggesting that other alleles of the glycophorin A-B-E region affect susceptibility to malaria. A functional study suggested that an allele at *GYPC* encoding Gerbich negative blood types, and at high frequency in Melanesians, was protective against *P. falciparum* invasion (Maier et al. 2003); however, there is no support for this from recent large-scale association studies in other populations.

Malaria is known to have been a major agent of natural selection in humans who live where malaria is endemic. Because of the role of glycophorins in malaria, there are several studies that assess genetic variation for signs of natural selection, and discover evidence for natural selection at the glycophorin A-B-E region (Baum et al. 2002; Ko et al. 2011; Bigham et al. 2018; Johnson and Voight 2018). Although this is consistent with our expectations, methods using sequence diversity and divergence may be biased because of the highly duplicated nature of the glycophorin A-B-E region, and the extensive recombination, copy number variation and gene conversion that occurs. More recent selection can be detected using an extended haplotype test, which compares LD with allele frequency to test for strong recent positive selection of a variant, and is likely to underestimate selection in the presence of gene conversion. The DUP4 variant is young as it is restricted to East Africa (Table 1, Leffler et al. 2017). Using the extended haplotype test, it has been shown that DUP4 has undergone recent positive selection to rapidly increase in frequency, presumably due to its protective effect against malaria (Leffler et al. 2017).

**Table 1:** DUP4 allele frequencies

**Country**	**Location**	**DUP4 allele frequency**	**Reference**
Tanzania	Nyamisati	0.13	Algady et al. (2018)
Malawi	Blantyre	0.039	Leffler et al. (2017)
Kenya	Kilifi	0.09	Leffler et al. (2017)
USA	Chicago, African-American	0.005	Unger et al. (1987)
South Africa	Cape region, admixed	0.011	Moores et al. (1992)

Unlike glycophorins A, B and E, the *GYPC* gene is not the result of a recent duplication, and lacks close paralogues. Comparative evolutionary studies are therefore more straightforward as the correct orthologue can be confidently identified, and analysis of genetic diversity is not affected by potential mis-mapping of sequence reads. Comparative analysis has shown that glycophorins C and D, encoded by *GYPC*, have undergone recent natural selection of the extracellular domain, strongly suggesting pathogen-mediated evolution (Wilder et al. 2009).

## Glycophorins in other infectious diseases

There is some evidence that glycophorins A and B act as receptors for other pathogens. *Babesia divergens*, which, like *Plasmodium*, is a member of the Apicomplexa phylum, is an eukaryotic intracellular parasite which can cause malarial-like symptoms in immunocompromised humans, uses glycophorins A and B to enter the RBC (Lobo 2005).

Some strains of *Escherichia coli* bind to glycophorin A on the surface of RBCs (Cooling 2015), and glycophorin A acts as the receptor to reoviruses, double stranded RNA viruses which include the rotavirus family. The single stranded RNA viruses encephalomyocarditis virus and hepatitis A also seem to use glycophorin A as a receptor for infection. Influenza viruses have been shown to interact with glycophorin A, and because influenza viruses cannot replicate in the anucleated RBC, it has been suggested that glycophorins act as decoy receptors diverting infection away from other tissues (Gagneux and Varki 1999).

## Conclusion

Glycophorins are major glycoproteins of the RBC surface, and are receptors for the malarial parasite *P. falciparum*. The region containing three paralogous 120-kb repeats, carrying the *GYPA*, *GYPB* and *GYPE* genes, has been generated by repeated rounds of duplication between 10 and 15 MYA, and shows extensive complex structural variation. One structural variant, DUP4, encodes the Dantu blood group antigen and is strongly protective against severe malaria. The role of genetic variation in the response to other pathogens that use glycophorins as receptors remains unclear.
